# Prior Infection Does Not Improve Survival against the Amphibian Disease Chytridiomycosis

**DOI:** 10.1371/journal.pone.0056747

**Published:** 2013-02-22

**Authors:** Scott D. Cashins, Laura F. Grogan, Michael McFadden, David Hunter, Peter S. Harlow, Lee Berger, Lee F. Skerratt

**Affiliations:** 1 James Cook University, School of Public Health, Tropical Medicine and Rehabilitation Sciences, Townsville, Queensland, Australia; 2 Taronga Conservation Society Australia, Herpetofauna Division, Mosman, New South Wales, Australia; 3 New South Wales Office of Environment and Heritage, Biodiversity Conservation Section, Queanbeyan, Australia; Smithsonian’s National Zoological Park, United States of America

## Abstract

Many amphibians have declined globally due to introduction of the pathogenic fungus *Batrachochytrium dendrobatidis* (*Bd*). Hundreds of species, many in well-protected habitats, remain as small populations at risk of extinction. Currently the only proven conservation strategy is to maintain species in captivity to be reintroduced at a later date. However, methods to abate the disease in the wild are urgently needed so that reintroduced and wild animals can survive in the presence of *Bd*. Vaccination has been widely suggested as a potential strategy to improve survival. We used captive-bred offspring of critically endangered booroolong frogs (*Litoria booroolongensis*) to test if vaccination in the form of prior infection improves survival following re exposure. We infected frogs with a local *Bd* isolate, cleared infection after 30 days (d) using itraconazole just prior to the onset of clinical signs, and then re-exposed animals to *Bd* at 110 d. We found prior exposure had no effect on survival or infection intensities, clearly showing that real infections do not stimulate a protective adaptive immune response in this species. This result supports recent studies suggesting *Bd* may evade or suppress host immune functions. Our results suggest vaccination is unlikely to be useful in mitigating chytridiomycosis. However, survival of some individuals from all experimental groups indicates existence of protective innate immunity. Understanding and promoting this innate resistance holds potential for enabling species recovery.

## Introduction

Over the past 40 years, amphibians across the globe have rapidly declined and are now the most threatened class of vertebrates, with at least one third of all species threatened with extinction [1]. Apart from habitat loss, the main cause of these declines is the emergence of the disease chytridiomycosis caused by the pathogenic fungus, *Batrachochytrium dendrobatidis* (*Bd*; [2]–[3]). Multiple lines of evidence indicate *Bd* has recently spread worldwide from an unknown origin via human-mediated transport into naive populations [4]–[7]. As a result, hundreds of susceptible species are feared extinct or have been reduced to small and vulnerable populations, often in well-protected and intact habitat.

Conservation biology aims to maintain or restore biodiversity, often by protecting the overall health of ecosystems. However, when specific threats to biodiversity occur in otherwise healthy ecosystems (e.g. chytridiomycosis), it is prudent to abate these threats directly [8]. Unfortunately, few tools are currently available to abate chytridiomycosis in the wild [9], limiting the options of wildlife managers to the captive management of species to prevent extinction [10], [11]. Where reintroduction programs have been attempted most struggle or completely fail due to the ongoing impact of chytridiomycosis [12], [13]. This is a familiar issue facing animal relocations, and reintroduction efforts in other taxa have been unsuccessful due to the failure to address the processes causing decline [14], [15].

A vaccination that provides protective immunity against *Bd* or reduces infectious burdens to a sublethal level could be used to improve survival rates in reintroduced frogs and important wild populations. Intensive vaccination could buy time for the evolution of resistance or tolerance to infection by maintaining population size and genetic diversity in the face of extirpation due to small population bottlenecks [9], [16]. Manipulating adaptive immunity through vaccination or inoculation has been successful in combating numerous diseases of domestic and wild animals as well as humans. Well-known examples of vaccination in wildlife include the control of sylvatic plague in black-footed ferrets [17] and the control of rabies in mammals [18]. It has been suggested that similar methods could be used to help control chytridiomycosis. Although there are no fungal vaccines yet approved for any animal, antibodies to various human fungal pathogens can be protective and promising trials are underway for *Candida* and *Cryptococcus* [19].

Amphibians, like all vertebrates, have a complex immune system, consisting of both innate and adaptive components [20]. Recent evidence suggests adaptive responses may be important in fighting chytridiomycosis [21], [22]. The African clawed frog (*Xenopus laevis*) produced a higher concentration of mucosal antibodies following exposure to *Bd*, however, it remains unknown, whether this is effective in controlling infection and improving survival and whether the response is heightened with re-exposure. Experimental elimination of splenic lymphocytes (via X-irradiation) of *X*. *laevis* resulted in greater infection intensities and decreased weight suggesting that the adaptive immune system may be important in controlling infection [23]. However, subcutaneous injection of heat (host: *X. laevis*) or formalin-killed (host: mountain yellow-legged frog; *Rana muscosa*) *Bd* did not control infection or improve survival even though it induced a systemic response in *X. laevis* resulting in circulating antibodies to *Bd* [23], [24].

Route of exposure and the nature of the antigen may be important in inducing an effective adaptive response to chytridiomycosis. Subcutaneous or intraperitoneal injection less effectively stimulate epidermal antigen presenting cells leading potentially to a systemic immune response, rather than targeting the skin where *Bd* infection naturally occurs [16]. Stimulating this epidermal response may require topical exposure to live *Bd* to stimulate the normal route of infection and ensure appropriate epitopes are available to be recognized by the immune system [25]. Alternatively, if *Bd* is able to evade [26] or suppress [27], [28] the host immune response then a prior infection will likely have little effect on survival and development of an effective vaccination strategy will be more complex. Although exposure to a live pathogen is a crude form of vaccination, it is inexpensive and could be applied immediately to species urgently requiring interventions to survive. In this study we test the hypothesis that a real infection followed by treatment to clear *Bd* will provide immunity and increase survival following a second exposure. We investigate this in captive-bred individuals of the critically endangered booroolong frog (*Litoria booroolongensis*), which is thought to have declined due to chytridiomycosis in areas of New South Wales (NSW), Australia [29].

## Materials and Methods

### Ethics Statement

The research protocols were approved by the James Cook University (A1408) and Taronga Conservation Society (5a/07/09) animal ethics committees.

### Captive Husbandry

To ensure all animals involved in this study had no prior exposure to *Bd*, we raised all experimental frogs from the captively bred spawn of confirmed *Bd* negative wild collected adults. Mature *L. booroolongensis* were collected from a 3 km section of the Retreat River in Abercrombie River National Park (34° 7′ 18.13″ S, 149° 38′ 4.98″ E) on the central west slopes of NSW with the permission of the New South Wales Office of Environment and Heritage. Egg masses were raised in gently aerated 32 L plastic tubs. At 14 d post-hatching, five groups of 40 tadpoles from each spawn were transferred to individual trays, flushed every 4 hr with filtered water. Water temperature was 20–21°C and each tray had one UVB emitting fluorescent tube and one daylight fluorescent tube set to a 10∶14 h light:dark cycle. We fed tadpoles frozen endive and a fish food flake mix *ad libitum*. At metamorphosis groups of 20 individuals were transferred to 20 L plastic aquariums flushed with fresh water daily. Frogs were fed calcium and multivitamin dusted crickets twice and once per week respectively and kept on a 12∶12 h light:dark cycle in addition to natural light through windows until they were 6 mo old when we randomly assigned each frog to a treatment group, waited 7 d to allow acclimation, and began the experiment.

### Culture of *Batrachochytrium dendrobatidis*

We isolated *Bd* from an adult *L. booroolongensis* captured along the same stretch of the Retreat River in Abercrombie National Park where the founding captive colony animals were sourced (AbercrombieNP-L.booroolongensis-09-LB-P7) with the permission of the New South Wales Office of Environment and Heritage. The culture was maintained at 20°C on TGHL agar plates [3]. To harvest zoospores for exposures, we flooded plates with 10 ml dilute salts solution (in mMol: KH2PO4 1.0, CaCl2.H2O 0.2, MgCl2.2H2O 0.1) for 20 minutes. Three separate counts were made with a haemocytometer and averaged, and the stock zoospore solution diluted with the dilute salts solution to 150,000 zoospores/ml.

### Diagnostic Polymerase Chain Reaction (PCR)

Throughout the experiment, frogs were swabbed with sterile, dry cotton tipped swabs (Medical Wire & Equipment Co MW100-100), and analysed following established protocols with a quantitative PCR TaqMan Assay that estimates the number of *Bd* zoospores present on the swab [30]–[32]. PCR reactions were run in triplicate and we considered a sample positive if at least one well returned a positive reaction to maximize sensitivity and maintain specificity [33]. Following swabbing, the mass (0.1 g) of each frog was recorded.

### Experimental Design

**Figure 1:**
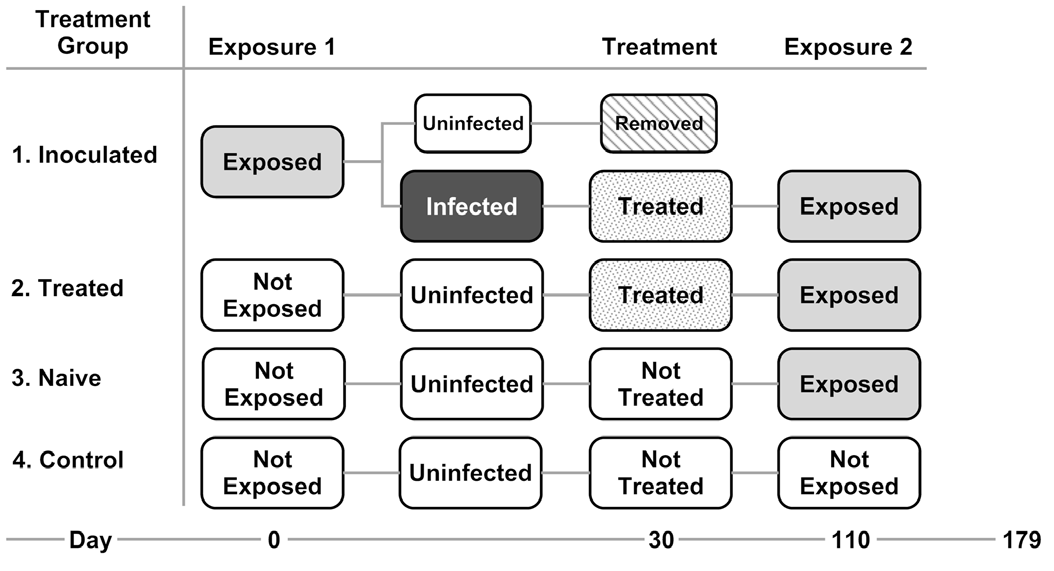
Flow chart of experimental treatment groups.

We established four experimental groups to investigate the effect of vaccination via pre-infection (Fig. 1). Group 1 (inoculated) was exposed to and infected with *Bd,* then cleared of infection with itraconazole and re-exposed to *Bd* 80 d post-treatment. Group 2 (treated) controlled for any residual antifungal effects of itraconazole and were not initially exposed to *Bd*, but were treated with itraconazole, then exposed to *Bd* for the first time. Group 3 (naïve) were not exposed, not treated and then exposed to *Bd* for the first time. Group 4 (control) were never exposed to *Bd* nor treated with itraconazole.

### Initial Exposure

We individually exposed 59 frogs to 750,000 zoospores in 5 ml dilute salts solution (sufficient to immerse the ventral surface of the frog) in 50 ml polyethylene tubs for 3 hrs on three consecutive days. At the end of each 3 hr exposure period and for 4 d following the last exposure, we transferred each frog and their inoculation broth to a 1000 ml polyethylene box containing 10 ml water (enough to cover the bottom of the container) that was replaced daily. We treated unexposed frogs similarly, but sham-exposed with only dilute salts solution. After 7 d, frogs were moved from the small exposure boxes to individual 10 L plastic aquariums fitted with a false bottom (plastic egg crate wrapped in shade cloth) for drainage, a hide, and a 250 ml polyethylene tub containing water. Room temperature was maintained at 20±2 °C, the same temperature at which the *Bd* cultures were grown, and within the temperature range when *L. booroolongensis* are most active in the wild. Sprinklers sprayed the inside of each aquarium with water for 1 min every 4 hr and water tubs were replenished daily. This setup maintained high humidity, but allowed frogs the choice to avoid standing water and partially dry. Frogs were swabbed at 0, 2, and 3 wks post-exposure. At 3 wk, 43/59 (73%) frogs were infected. One frog died with clinical signs of chytridiomycosis between weeks 3 and 4. We removed the 16 exposed frogs that did not become infected from the experiment as we were interested in the protective effect of a prior active infection, not just prior exposure, on subsequent exposure to the pathogen.

### Antifungal Treatment

At 4 wk, we began treatment to clear infection. Studies in the model amphibian *X. laevis* indicate T-cell mediated responses begin approximately 14 d post exposure [16]. Therefore 4 wks was considered sufficient time for priming an adaptive response via clonal expansion. We placed all 43 infected frogs (inoculated) and 11 unexposed frogs (treated) in individual 50 ml polyethylene tubs and poured 5 ml (enough to immerse the ventrum) 0.01% itraconazole (Sporanox) over the dorsal surface [34]. Frogs were kept in the treatment solution for 5 min daily for 7 d, before being returned to their aquaria. All frogs survived and cleared infection as indicated by three negative PCR results at 2, 5 and 11 wks following the end of treatment.

### Second Exposure

At 80 d post-treatment (109 d post 1^st^ exposure) we exposed frogs in the inoculated, treated and naive groups to 750,000 zoospores in dilute salts solution daily for 3 d as described previously. The control group was sham-exposed to dilute salts solution alone. We swabbed all frogs at 2, 3, 5 and 9 wk post-exposure and at death. At 70 d following the second exposure (179 d post 1^st^ exposure) all surviving frogs were euthanized with an overdose of MS-222 (2 g/L in water).

## Results

Prior *Bd* infection or prior treatment with itraconazole had no significant effect (α = 0.05, 2-sided Fisher’s exact test) on survival (p = 1.0), infection status (p = 0.058), proportion of infected individuals that cleared infection (p = 0.924) or the proportion of survivors that were infected at the end of the experiment (p = 0.487). Approximately half of the infected frogs in each treatment group successfully cleared infection and approximately 85% of frogs in each treatment group survived (Table 1).

**Table 1:** Infection, clearance and survival rates of each treatment group (excluding unexposed controls) following exposure to *Batrachochytrium dendrobatidis* (*Bd*).

	Inoculated	Treated	Naïve	Fisher’s exact test (p-value)
*Bd* infection rate (overall)	20/32 (63%)	10/11 (91%)	14/28 (50%)	0.058
*Bd* clearance rate	11/20 (55%)	5/10 (50%)	6/14 (43%)	0.924
Survival rate	27/32 (84%)	9/11 (82%)	24/28 (86%)	1.000
*Bd* infection rate (survivors)	4/27 (15%)	3/9 (33%)	4/24 (17%)	0.487
*Bd* infection rate (non survivors)	5/5 (100%)	2/2 (100%)	4/4 (100%)	

“Inoculated” frogs were previously exposed and infected with *Bd* and cleared of infection with itraconazole prior to exposure, “Treated” frogs were not initially exposed to *Bd* but were treated with itraconazole prior to exposure, “Naïve” frogs were not exposed nor treated prior to exposure.

**Figure 2:**
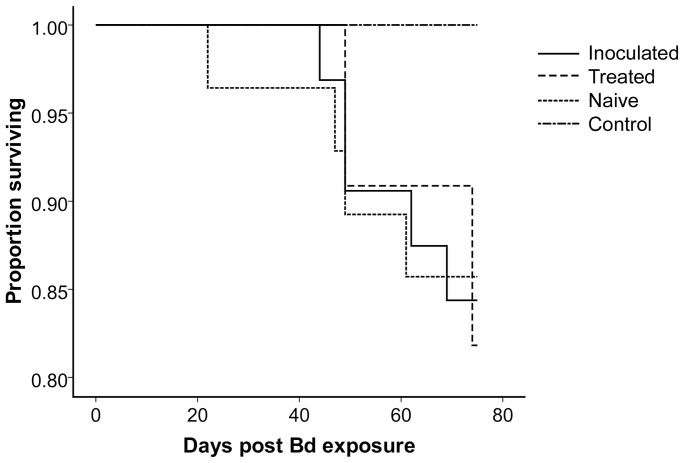
Kaplan-Meier curve depicting the proportion of frogs surviving in each treatment group over the days post Bd exposure. “Inoculated” frogs were previously exposed and infected with Bd and cleared of infection with itraconazole prior to exposure. “Treated” frogs were not initially exposed to Bd but were treated with itraconazole prior to exposure. “Naïve” frogs were not exposed nor treated prior to exposure. “Control” frogs were never exposed nor treated at any point during the experiment.

A Kaplan-Meier survival curve using days survived as the response variable and censoring individuals that survived until the end of the experiment revealed no significant difference among groups using either the log-rank or Wilcoxon tests (Fig. 2, p>0.22 for both tests), and a Cox proportional hazards regression showed that neither experimental group, sex nor mass had a detectable effect on survival. All experimental groups continued to feed normally and increased in body mass following the second exposure. A one-way analysis of variance (ANOVA) revealed no significant difference in change of body mass among treatments between the second exposure and the end of the experiment (ANOVA; df = 3, F = 2.34, p = 0.083) indicating lack of a strong sublethal effect on body condition.

**Figure 3:**
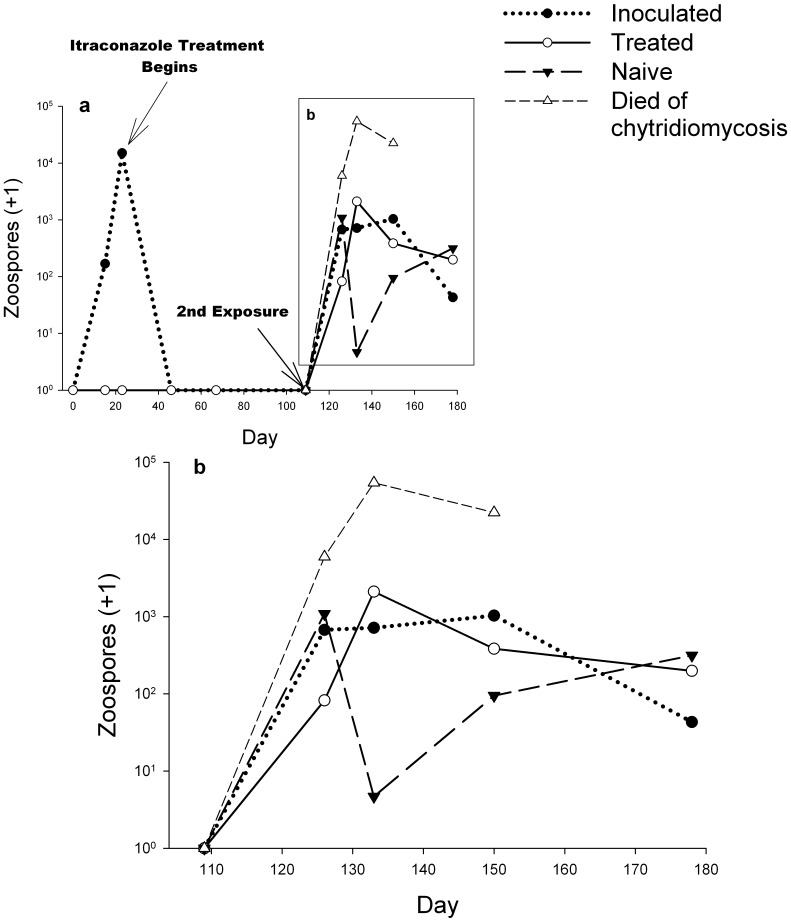
Mean intensity of *Batrachochytrium dendrobatidis* infection over time. a) Initial exposure of and treatment of “inoculated” frogs and treatment of “treated” frogs. b) Second exposure of all frogs except unexposed controls (controls not shown). Frogs that died of chytridiomycosis are from across treatment groups. All other lines depict only frogs that survived.

All frogs that died during the experiment were infected (Table 1) and their mean intensity of infection was significantly higher at each sampling period than in infected frogs surviving to the end of the experiment (Fig. 3). In infected survivors, mean infection intensity increased rapidly within the first 2 wks following the second exposure and then plateaued (Fig. 3). Possible differences in infection intensity over time (beginning 2 wks post re-exposure) among treatments were analysed with a factorial, repeated measures ANOVA using Log10 transformed PCR results of frogs that were infected at least once during the experiment but did not die. Degrees of freedom were corrected using Greenhouse-Geisser estimates as the data violated the assumption of sphericity. Neither the interaction of treatment and time (*F*(4.5,67.7) = 1.9, p = 0.113) nor time (*F*(3,67.7) = 0.82, p = 0.457) or treatment alone (*F*(2,30) = 2.76, p = 0.079) had a significant effect on intensity of infection (Fig. 3).

## Discussion

We found no evidence for increased protective immunity following infection with *Batrachochytrium dendrobatidis*. Prior exposure and treatment of *Bd* has been widely promoted as a straightforward and inexpensive, but untested vaccination method to improve the survival of susceptible species [21], [25], [35]. Our results show, however, that prior infection and treatment is unlikely to be an effective management strategy for *L. booroolongensis*, and possibly other species threatened by chytridiomycosis worldwide. While the relatively high survival and moderate cure rates indicate many of the frogs in this study had some form of resistance, the inability of a prior infection to increase protective immunity is striking, and a major blow to conservation efforts.

Although we found a near significant effect of treatment on prevalence (Fisher’s exact test: p = 0.058) and intensity of infection (Repeated Measures ANOVA: *F*(2,30) = 2.76, p = 0.079), the direction of the effect for both results was opposite what would be expected with a successful vaccination. If prior infection provides a benefit, the ‘inoculated’ frogs should have lower prevalence and mean intensity of infection than the other experimental groups. Instead, prevalence was similar between the ‘inoculated’ and ‘naïve’ groups but higher in the ‘treated’ group (Table 1), and intensity of infection was similar across all treatments and time periods, but lower in the ‘naïve’ group 3 wk post re-exposure (Fig. 3). Neither of these unexpected and marginally significant results has a clear biological explanation, thus we decline from further speculation about their cause.

It remains unclear why prior exposure was ineffective against *Bd*. It is possible that Bd avoids and down regulates the adaptive immune response. Fungal diseases of amphibians typically incite strong inflammatory reactions observed microscopically [36], but this response is not observed in skin infected with *Bd* [37]. Berger et al. (1999) [38] suggested that sporangia (the reproductive phase of *Bd*) may effectively evade recognition by the host due to their location inside cells of the superficial epidermis. Intracellular and epithelial sites of infection are both features in common with other fungi that can hide from phagocytes [26]. In addition to evading the amphibian immune system, recent evidence suggests *Bd* may also actively suppress an immune response. In the clawed frog *Silurana tropicalis*, infection results in down regulation of genes associated with toll-like receptors (which could assist pathogen recognition), complement pathways, and B and T-lymphocytes [27], [28]. This is possibly due to the release of soluble factors inhibitory to B and T-cells as demonstrated by in-vitro lymphocyte proliferation assays (J. Ramsey et al., unpublished in Rollins-Smith et al. 2011 [35]). General mechanisms of fungal evasion and suppression include shielding surface antigens, inducing anti inflammatory cytokines, decreasing inflammatory cytokines and phenotypic switching. The ability to evade or inhibit the immune system is a feature of other highly virulent diseases that, like chytridiomycosis, can kill previously healthy hosts and are difficult to control, e.g. malaria.

Although potential remains to develop a targeted *Bd* vaccine that overcomes possible evasion and inhibition of the adaptive immune response, it will likely be too expensive and lengthy an exercise to help critically threatened amphibian populations in the near future. For example, it has taken two decades and $300 million in investment to develop an effective malaria vaccine that cuts the risk of infection in infants by 56% [39], [40].

Despite the current unsuitability of prior exposure as a vaccination strategy, our findings contribute to a broader understanding of the ecology of chytridiomycosis. The lack of an observable adaptive response to *Bd* (absence of an ‘immune’ state) helps explain the dynamics observed in the field, such as the regular re-infection of individuals [41], [42] and annual seasonal outbreaks [3], [7], [36], [43]–[44].

Although we found no evidence that prior infection elicits an effective adaptive immune response, the majority of booroolong frogs in this study clearly had some form of resistance. Despite inoculation with 750,000 zoospores for 3 d (a dose higher than would be experienced in the wild), 38% of frogs never developed a detectable infection. Of the 62% that were infected, mortality rates were relatively low (25%) across experimental groups, and in surviving frogs infection intensity peaked 3 wk post exposure before stabilising or declining for all treatments. Overall, 50% of all infected frogs successfully cleared infection, 25% maintained a sublethal infection until the end of the experiment and 25% were unable to control pathogen replication and died with elevated zoospore burdens.

Mortality from chytridiomycosis in the field has been reported to occur once the infectious burden increases above a mean threshold of 10^4^–10^5^ zoospore equivalents [42], [44]. A similar fatal threshold was found in this study and individuals able to eliminate or maintain infection below 10^4^–10^5^ zoospores survived. Frogs were housed in optimal conditions for the pathogen (20°C and high humidity [45]) throughout the experiment, eliminating the possibility of experimental conditions alone causing clearance of infection. However, frogs were given the freedom to avoid direct contact with standing water (as would occur in the wild for this species) which could help limit pathogen proliferation and re-infection.

As observed resistance was unrelated to prior exposure, innate immune responses are likely responsible for limiting pathogen replication below the threshold. Interestingly, this resistance was not apparent when we exposed booroolong frogs to the same *Bd* zoospore dose in groups of 10 (750,000 zsps/10 frogs) and 100% of frogs died due to chytridiomycosis (compared with 100% survival in unexposed controls; S. Cashins unpublished data), suggesting defences are overwhelmed with constant re-exposure from nearby hosts. This supports field studies identifying population density as an important predictor of *Bd* infection intensity and impact [42], [44] and may help explain why *L. booroolongensis* have declined in the wild despite their moderate susceptibility in captivity.

Vaccination via live infection has been a commonly proposed solution to mitigate the impact of chytridiomycosis on amphibian biodiversity [9]. Although our study did not support this as an effective strategy, a number of potential management solutions remain which are aimed at either the pathogen or the host.

Pathogen-focused strategies involve intensive site management such as the manipulation of water chemistry or natural chytrid predators to reduce pathogen survival and density in the environment [46], [47]. These strategies hold the most promise at select, discrete pond sites of high conservation value where ongoing and intensive human involvement is available to maintain conditions inhospitable for *Bd*.

Host-focused strategies seek to promote the factors responsible for host persistence. Some species that suffered range contractions due to *Bd* are now recolonising previously inhabited areas, suggesting one or more of three possibilities: 1. resistance is evolving naturally. 2. *Bd* is becoming less virulent and 3. lower density populations are able to survive in areas were higher population densities were extirpated [48], [49]. Species at high risk of extinction may thus be effectively managed if the underlying mechanisms of host resistance, pathogen virulence and disease dynamics are identified.

The potential for manipulating pathogen virulence has yet to be explored and while limiting host-density may provide a short term solution to species survival it is counterproductive to conservation in the long-term. Increasing the reproductive fitness or distribution of individuals with more robust innate immunity through headstarting, translocation of survivors, or reintroduction programs that select for resistance currently holds the most promise for long-term mitigation of disease in species threatened with chytridiomycosis. Differences in susceptibility between and within species have been described in this and other studies [50], [51], and have been correlated with individual aspects of innate immunity such as antimicrobial peptides [52], MHC diversity [22] or competitive skin bacteria [53]. However, *Bd* is an unusual pathogen (as the only member of the Phylum Chytridiomycota to cause disease in vertebrates and as a fatal cutaneous fungus) and so host immune mechanisms may also be unusual and complementary. Studies that adopt a broad, exploratory approach and investigate multiple components of innate immunity through immunological, genetic and post-genomic analyses are needed to begin identifying the most important mechanisms of observed resistance.

Many endangered amphibian species threatened by chytridiomycosis exist as small populations that are unsustainable. We have found that vaccination via pre-exposure with live *Bd* is unlikely to be of assistance.
